# *In-utero* Exposure to Maternal Stressful Life Events and Risk of Cryptorchidism: The Raine Study

**DOI:** 10.3389/fendo.2019.00530

**Published:** 2019-08-02

**Authors:** Elvira V. Bräuner, Martha Hickey, Åse Marie Hansen, Dorota A. Doherty, David J. Handelsman, Anders Juul, Roger Hart

**Affiliations:** ^1^Department of Growth and Reproduction, Rigshospitalet, University of Copenhagen, Copenhagen, Denmark; ^2^The International Research and Research Training Centre in Endocrine Disruption of Male Reproduction and Child Health, Rigshospitalet, University of Copenhagen, Copenhagen, Denmark; ^3^Department of Obstetrics and Gynaecology, University of Melbourne, Melbourne, VIC, Australia; ^4^Department of Public Health, University of Copenhagen, Copenhagen, Denmark; ^5^The National Research Centre for the Working Environment, Copenhagen, Denmark; ^6^Division of Obstetrics and Gynaecology, University of Western Australia, Perth, WA, Australia; ^7^ANZAC Research Institute, University of Sydney, Sydney, NSW, Australia; ^8^Fertility Specialists of Western Australia, Bethesda Hospital, Claremont, WA, Australia

**Keywords:** pregnancy, stressful life events, *in-utero* exposures, cryptorchidism, Raine study

## Abstract

Cryptorchidism, registered at birth or later, is the most common birth defect in males in western countries, estimated to affect around 2–3% of newborn boys, declining to around 2% at 3 months. We have previously described a potential association between stressful life events (SLEs) in pregnancy and reduced semen quality and testosterone levels in adult offspring. Both outcomes are believed to share a common etiology with cryptorchidism thus increased risk of cryptorchidism in boys exposed to prenatal SLEs may be plausible. The risk of cryptorchidism associated with prenatal SLE amongst 1,273 male Generation 2 offspring was estimated using the Western Australian Pregnancy (Raine) Study. SLEs are discrete experiences that disrupt an individual's usual activities causing a life change and readjustment, such as death of a relative or friend, divorce, illness or job loss. Mothers prospectively reported SLEs, during pregnancy at gestational weeks (GW) 18 and 34 using a standardized 10-point questionnaire. A boy was diagnosed as cryptorchid if one or both testes was non-palpable in the scrotum and not able to be manipulated into the scrotum. Twenty-four (2%) cryptorchid boys were identified. Mean (standard deviation) of SLE exposures in GW34 was 1.1 (1.2) for non-cryptorchid boys and slightly higher 1.5 (1.8) for cryptorchid boys, similar differences were observed in GW18. Adjusted odds ratio [OR] and 95% confidence intervals (CI) for risk of cryptorchidism in early (18-weeks) and late gestation (34-weeks) according to prenatal SLE exposures were: 1.06 (95% CI: 0.77–1.45) and 1.18 (95% CI: 0.84–1.67), respectively. This is the first-time report on the possible relationships between exposure to early and late pregnancy SLEs and risk of cryptorchidism in a birth cohort. Prenatal SLE exposure was not associated with a statistically significant increase in the risk of cryptorchidism in male offspring. A small case population limits the statistical power of the study and future larger studies are required to evaluate this potential association.

## Introduction

Cryptorchidism is the most common anomaly in males in western countries and is estimated to affect around 2–3% of full-term newborn boys, declining to around 2% at 3 months (1). The etiology of cryptorchidism has not been fully elucidated, but a variety of risk factors have been reported including defects in specific genes, prematurity, fetal growth restriction and low parity/birthweight (2). There is also growing support for a possible role of prenatal exposures, such as maternal alcohol and smoking (3, 4) and it has been proposed that disorders of the male reproductive tract may have a common etiology during fetal life, described as the testicular dysgenesis syndrome (5, 6). Acquired cryptorchidism after birth in previously scrotal testes is now also well-recognized, although acquired cryptorchidism is less studied this event has been reported to affect 0.2% boys at ages 3 months and 0.6% of boys assessed at both 18 and 36 months (7). The precise etiology of acquired cryptorchidism is not yet known but mechanical as well as endocrine factors are believed to play an important role (8).

Multistage testicular descent, starting in gestational week (GW)8 continues until approximately GW35, indicating that testicular descent might be sensitive to adverse exposures throughout the entire pregnancy (9, 10). Normal testicular development and descent in humans is also strongly dependent on reproductive hormones and exposure to environmental chemicals or pharmaceuticals has been proposed to disrupt these programming mechanisms in the fetus (3). However, non-chemical exposures, including stressful life events (SLEs), may also disrupt the prenatal hormonal milieu as well and gestational exposure to SLEs has been identified as having deleterious reproductive effects on human male fetuses (11). This is supported by animal studies which suggest that stressors (such as prenatal restraint of the animal) during pregnancy can affect reproductive function in male offspring including delayed testicular descent (12–15).

Presently, very limited human evidence exists regarding the role of the emotional and psychological state of the pregnant women due to SLEs. Given the sensitivity of male fetal genital development to external factors it is plausible that prenatal exposure to maternal SLEs during pregnancy may impact the risk of cryptorchidism in offspring, but no previous studies have investigated this in a cohort setting. We have previously described a potential association between exposure to maternal SLEs in pregnancy and reduced semen quality and testosterone levels in the male offspring in adulthood (16), we thus hypothesize that maternal SLE exposures may also increase the risk of cryptorchidism as these outcomes are considered symptoms of disrupted development of the reproductive system (3, 17). This present paper presents the results of the prospective examination of the association between the number of maternal SLEs in both early and late gestation and risk of cryptorchidism in the male Generation 2 offspring in the Western Australian Pregnancy Cohort (Raine) Study.

## Methods

### The Raine Study

**Figure 1:**
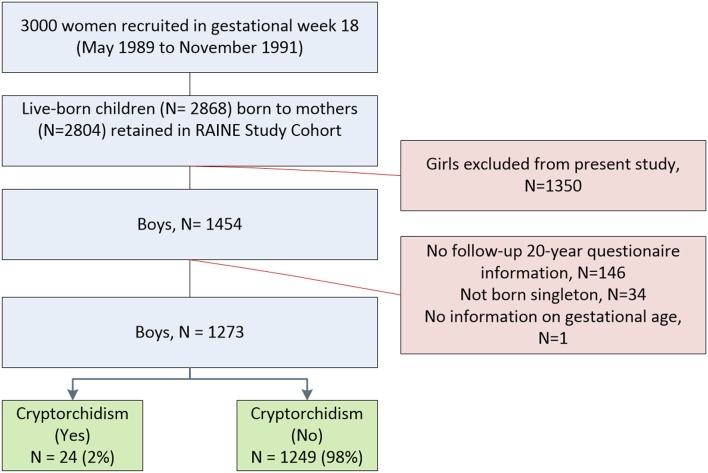
Study population and exclusions.

The Western Australian Pregnancy Cohort (Raine) Study, formed from a pregnancy cohort study, was designed to measure the relationships between early life events and subsequent health and behavior. The study recruited almost 3,000 women in GW18 within the period from May 1989 to November 1991. 2,868 children (including 1,454 boys) live-born children and their mothers were retained to form the Raine Study cohort (18). Of the 1,454 liveborn Generation 2, 146 did not have complete data on prenatal SLE exposure questionnaires, and we excluded boys not born singleton (*n* = 34), and boys with missing information on gestational age (*n* = 1), leaving 1,273 sons for inclusion (Figure 1).

### Exposure Variable

#### Stressful Life Events (SLEs)

Data on the SLEs experienced by each woman were collected at 18- and 34-weeks' gestation using a 10-item questionnaire based on the validated Tennant and Andrews' Life Stress Inventory (19, 20). SLEs included death of a close relative, death of a close friend, separation or divorce, marital problems, problems with children, own involuntary job loss, partner's involuntary job loss, money problems, pregnancy concerns, residential move and other problems (Table 1). Women were asked to record individual SLEs since the confirmation of pregnancy at 18 weeks' gestation, and at 34 weeks they were asked about SLEs in the previous 4 months of pregnancy to ensure that the same SLE was not counted twice. Each response to each of the 10-item questionnaire were recorded as “yes/no” once, in accordance with previous studies to maximize recall (21). There was a minimum to maximum of zero to 10 SLEs per women in each gestational survey.

**Table 1:** Type, timing, and number of stressful life event (SLE) exposures of 1,273 sons according to cryptorchidism.

**Stress life event (SLE)^a^**	**GW18 only**, ***N*** **(%)**			**GW34 only**, ***N*** **(%)**		
	**Cryptorchidism (No, *n* = 1249)**	**Cryptorchidism (Yes, *n* = 24)**	***P*-value^b^**	**Cryptorchidism (No, *n* = 1249)**	**Cryptorchidism (Yes, *n* = 24)**	***P*-value^b^**
**Individual SLE**						
Death of a relative	75 (6.0)	4 (16.7)	0.03	64 (5.1)	2 (8.3)	0.48
Death of a friend	24 (1.9)	1 (4.2)	0.43	30 (2.4)	0 (0)	0.44
Your own job loss (not voluntary)	33 (2.6)	0 (0)	0.42	25 (2.0)	1 (4.2)	0.46
Your partner's job loss (not voluntary)	61 (4.9)	2 (8.3)	0.44	70 (5.6)	4 (16.7)	0.02
Pregnancy concerns	319 (25.5)	4 (16.7)	0.32	251 (20.1)	6 (25.0)	0.55
Separation or divorce	46 (3.7)	1 (4.2)	0.91	33 (2.6)	0 (0)	0.42
Residential move	191 (15.3)	6 (25.0)	0.19	236 (18.9)	4 (16.7)	0.78
Marital problems	109 (8.7)	4 (16.7)	0.18	89 (7.1)	3 (12.5)	0.31
Problems with your children	80 (6.4)	2 (8.3)	0.70	80 (6.4)	3 (12.5)	0.23
Money problems	333 (26.7)	5 (20.8)	0.52	314 (25.1)	11 (45.8)	0.02
Other problems	197 (15.8)	3 (12.5)	0.66	136 (10.9)	1 (4.2)	0.29
*N* (total individual SLEs reported)	1,468	32		1,328	35	

a *The questionnaire at GW18 related to the period since becoming pregnant, and on the GW34 questionnaire, the women were asked whether any of the SLEs had been experienced during the past 4 months, ensuring that the same event was not counted twice*.

b *Chi^2^ test for SLE exposure amongst cryptorchid/non-cryptorchid boys*.

#### Number and Timing of SLEs

A-priori evidence on the central role of prenatal exposures (3, 4) and the multistage testicular descent, which starts in GW8 and is reported to continue until approximately GW35, indicates that testicular descent is sensitive to adverse exposures throughout the entire pregnancy (9, 10). Thus, we created a continuous variable defined as the total number of SLEs reported at 18- and 34-weeks gestational surveys, weighting each SLE equally.

### Cryptorchidism

All children were followed-up thoroughly for dysmorphology of all organs including the genitals at 1 year of age. Examination of the male genitals was performed by a trained pediatrician and took place in a warm room with the boy in the supine position. Cryptorchidism was ascertained by standard methods of testis manipulation in the scrotum using warm hands. The boy was diagnosed as cryptorchid (congenital and acquired) if one or both testes was non-palpable within the scrotum or not able to be manipulated into the scrotum.

### Statistical Strategy

Frequency counts for exposures, co-variates and cryptorchidism were computed and logistic regression analysis was applied to estimate the risk of cryptorchidism according to prenatal SLE exposures. The effects of timing of the SLE exposure were assessed by separately examining the effects of early gestational SLE exposures (reported at GW18) and late gestational SLE exposure (reported at GW34). When addressing effects of SLEs occurring in late gestation (GW34), we mutually adjusted all models for SLEs reported at GW18. Crude and adjusted ORs were estimated.

Based on the literature supporting an association with maternal stress and risk of cryptorchidism or based on differences in levels of the co-variate between cryptorchid and non-cryptorchid boys, the following co-variates were included in the adjusted models: sons birthweight (< or ≥2,500 g), gestational age at birth (< or ≥37 weeks), parity, maternal age (years), lifestyle (alcohol consumption (yes/no), smoking (yes/no), socioeconomic status as reported by the mother [total household annual income: dichotomized to reflect a minimum income level (< AUD 24,000 p.a. or ≥AUD 24,000 p.a.) according to the Australian Government guidelines at the time of the pregnancies (1989–1991)] and mothers education (none vs. trade/non-degree, college/university or other). The statistical power of the study to detect an OR of 1.50 at the 0.05 significance level was 52%.

Odds ratios (OR) and 95% confidence intervals (CI) (95%) reflect the risk per increment increase in exposure to prenatal SLEs. None of the 1,273 boys in the included population were born before week 24 or after week 43, nor did any of the included boys have a birth weight of <500 g or above 8,000 g (considered extreme outliers). All statistical analyses were performed by SAS, using the LOGISTIC procedure (version 9.4; SAS Institute, Cary, NC version 9.4).

The Raine study was approved by the University of Western Australia Human Research Ethics Committee consistent with the Declaration of Helsinki and written informed consent was obtained from all participants prior to enrollment. The study is reported according to the STROBE (Strengthening the Reporting of Observational Studies) guidelines and checklist.

## Results

Of 1,273 included boys, 24 (2%) were registered with cryptorchidism (congenital and acquired). Overall, money problems (20.8–45.8%) were the most frequently reported individual SLE exposures in both gestational periods followed by pregnancy concerns (16.7–25.5%) and residential move (15.3–25.0%). A significantly higher proportion of cryptorchid boys were exposed to the SLE “Death of a relative” reported at GW18 as well as “partners involuntary job loss” and “money problems” reported in the GW34 (Table 1).

The characteristics of the mothers and sons (overall and stratified according to cryptorchid status) are reported in Table 2. Mean (standard deviation, SD) of SLE exposures in GW18 was 1.3 (1.5) for cryptorchid boys and slightly lower 1.2 (1.2) for non-cryptorchid boys. The same was observed in GW34 with 1.5 (SD: 1.8) mean SLE exposures for cryptorchid boys and lower mean SLE exposures for non-cryptorchid boys 1.1 (SD: 1.2). The mothers of boys with cryptorchidism were younger, consumed less alcohol, were less likely to be current smokers, had lower family incomes, but more likely to have an education and to have given birth to at least one previous child than the mothers of non-cryptorchid boys. Cryptorchid boys were more likely to have birthweights below 2,500 g and be born preterm. Table 2 also shows the results of the logistic [crude and adjusted (cf. statistical strategy)] regression analyses amongst the 1,273 boys. The adjusted odds ratio [OR] and 95% confidence intervals (CI) for risk of cryptorchidism in early (18-weeks) and late gestation (34-weeks) according to prenatal SLEs were: 1.06 (95% CI: 0.77–1.45) and 1.18 (95% CI: 0.84–1.67), respectively. The *post-hoc* calculated statistical power of the study to detect OR of this magnitude at the 0.05 significance level was 15%.

**Table 2:** Characteristics of the 1,273 sons and association between SLE exposure in early (GW18) and late (GW34) pregnancy and risk of cryptorchidism in sons.

	**All/*N* (%) or Average (SD)**	**Cryptorchidism/*****N*** **(%) or Average (SD)**		**Crude OR (95% CI)^f^**	**Adjusted model OR (95% CI)^g^**
**SLE exposure**^a^		Yes (*n* = 24)	No (*n* = 1249)		
GW18	1.18 (1.21)	1.33 (1.49)	1.18 (1.21)	1.11 (0.81; 1.51)	1.06 (0.77; 1.45)
GW34	1.08 (1.20)	1.46 (1.82)	1.06 (1.18)	1.27 (0.92; 1.77)	1.18 (0.84; 1.67)
**Lifestyle- and person-related characteristics of mothers**^b^					
Maternal age at pregnancy (<25 years)	402 (30.7)	8 (33.3)	382 (30.6)	0.99 (0.92; 1.06)	0.99 (0.92; 1.07)
Consumed alcohol (yes)^b^	617 (47.2)	11 (45.8)	591 (47.3)	0.94 (0.42; 2.12)	1.14 (0.49; 2.67)
Smoker (yes)^b^	322 (24.6)	5 (20.8)	313 (25.1)	0.79 (0.29; 2.13)	0.60 (0.21; 1.70)
Low family income^c^	596 (45.6)	15 (62.5)	571 (45.7)	1.72 (0.91: 3.22)	1.81 (0.73: 4.53)
Education (None)^d^	672 (51.4)	10 (41.7)	609 (48.8)	1.33 (0.59; 3.02)	1.23 (0.51; 2.95)
Parity (≥1)	686 (52.5)	15 (62.5)	651 (52.1)	1.53 (0.67; 3.52)	1.66 (0.66; 4.18)
**Person-related characteristics of sons**					
Birthweight (<2,500 g)	94 (7.2)	3 (12.5)	63 (5.0)	2.60 (0.76; 8.95)	0.77 (0.15; 3.91)
Gestational age at birth (<37 weeks)	67 (5.2)	5 (20.8)	66 (5.3)	4.72 (1.71; 13.0)	5.58 (1.47; 21.3)
Cryptorchidism (yes)^e^	24 (1.9)	24 (100)	–	–	–

a *The questionnaire at GW18 related to the period since becoming pregnant, and on the GW34 questionnaire, the women were asked whether any of the events had been experienced during the past 4 months, ensuring that the same event was not counted twice*.

b *As reported by mothers in questionnaire data at gestational week 18*.

c *Average annual family income level per annum below AUD 24,000 reflecting the minimum income level in 1989–1991, according to the Australian Government guidelines*.

d *No education vs. some form of education including: trade, non-degree, college degree, university degree, or other education*.

e *Includes both unilateral and bilateral cryptorchidism*.

f *Each exposure variable or co-variate included individually in the model, except for the model estimating the risk of SLE exposures in GW34 which was adjusted for exposure in GW18*.

g *The fully adjusted OR for each SLE exposure period, and mutually adjusted OR for each co-variate in the risk model for both gestational periods (GW18 + GW34)*.

## Discussion

This is the first study using a birth cohort to examine the prospective relationships between exposure to early and late gestational SLEs and risk of cryptorchidism in male offspring. Prenatal SLE exposure was not associated with a statistically significant increase in the risk of cryptorchidism in male offspring.

In a recent study we reported that maternal SLEs were potentially associated with reduced semen quality and serum testosterone levels in adult sons (16) and in this present study we aimed to investigate whether that relationship could be explained by an increased prevalence of cryptorchidism. A significant relationship might have explained the hypothesis that disorders of the male reproductive tract may have a common etiology during fetal life, described by Sharpe and Skakkebæk as the testicular dysgenesis syndrome (6), however, the number of cryptorchid cases in the present study did not allow this hypothesis to be fully tested, as only 24 boys were registered with cryptorchidism, limiting statistical power.

No previous epidemiological studies have comprehensively assessed the association between total SLE exposures in pregnancy and risk of cryptorchidism in offspring in a birth cohort setting. One previous registry-based study has been published, focusing only on maternal bereavement during pregnancy and reproductive function in sons. That study included all males born in Denmark between 1973 and 2008 (*n* = 1,217,576) and in sub-analyses the association between prenatal bereavement in both first and second trimester and risk of cryptorchidism in sons, registered at birth in the Danish National Patient Registry was reported to be positive and significant, supporting the premise and results of our present study (22). However, another registry-based study also assessing bereavement during pregnancy and the risk of cryptorchidism at birth reported no associations (23). Both previous studies only considered one SLE, namely bereavement due to death of a close relative, and other unmeasured SLEs in those studies may have introduced some residual confounding. Also, the cryptorchidism diagnoses from the register data in those studies would include boys with undescended testis at birth that later descend spontaneously and would also not account for boys with acquired cryptorchidism, leading to some random misclassification (24). Future larger studies evaluating the effect of multiple gestational SLEs are required to evaluate this potential effect.

### Strengths and Limitations

The strengths of the present study include the detailed prospective collection of data from early and late gestation about maternal SLEs. But on the other hand, these reports of maternal SLEs are subjective, implying a potential variation in coping and perceptions of stress and the prevalence of SLEs reported. The serious nature of SLEs implies minimal risk of recall bias, but some women may have forgotten to report an SLE that was not specifically listed on the 10-point questionnaire possibly introducing some bias. Another strength of the study was the inclusion of important risk factors for cryptorchidism and/or potential confounders including birthweight, premature birth, parity and maternal age, lifestyle, education, and socioeconomic status. Finally, confirmed cryptorchidism at age 1 year by a trained medical practitioner, implies less risk of misclassification, as many boys registered with undescended testis at birth, will later have testis that descend spontaneously; also, other boys with acquired cryptorchidism are included in the ascertainment. We do not expected the inclusion of boys with acquired cryptorchidism to affect our result as both congenital and acquired cryptorchidism are thought to be sensitive to disruption of the prenatal hormonal milieu (8, 12–15), which was the focus of our present study.

A major limitation of the present study is the small case population which limited the statistical power, and possibly implied risk of a false negative result (Type II error). Thus, larger prospective studies are needed, as it was not feasible to increase sample size within the Raine study.

## Conclusion

Prenatal SLE exposure was not associated with a statistically significant increase in the risk of cryptorchidism in male offspring. A small case population limited the statistical power of the study and future larger studies are required to evaluate this potential association.

## Data Availability

All datasets generated for this study are included in the manuscript/supplementary files.

## Ethics Statement

The studies involving human participants were reviewed and approved by University of Western Australia Human Research Ethics Committee consistent with the Declaration of Helsinki and written informed consent was obtained from all participants prior to enrollment.

## Author Contributions

EB drafted the manuscript and performed statistical analyses. AJ, ÅH, RH, MH, and DD contributed to the manuscript preparation. EB, AJ, and RH contributed to the concept and design for the study. RH and DD provided all the Raine Study data. DD prepared data for analyses and collaborated with EB on the statistical strategy. All authors contributed to critical interpretation of data and the final draft of the manuscript.

### Conflict of Interest Statement

The authors declare that the research was conducted in the absence of any commercial or financial relationships that could be construed as a potential conflict of interest.
